# The Role of *GJD2*(Cx36) in Refractive Error Development

**DOI:** 10.1167/iovs.63.3.5

**Published:** 2022-03-09

**Authors:** Emilie van der Sande, Annechien E. G. Haarman, Wim H. Quint, Kirke C. D. Tadema, Magda A. Meester-Smoor, Maarten Kamermans, Chris I. De Zeeuw, Caroline C. W. Klaver, Beerend H. J. Winkelman, Adriana I. Iglesias

**Affiliations:** 1 Department of Ophthalmology, Erasmus Medical Center, Rotterdam, The Netherlands; 2 Department of Epidemiology, Erasmus Medical Center, Rotterdam, The Netherlands; 3 Netherlands Institute for Neuroscience (NIN), Royal Dutch Academy of Art & Science (KNAW), Amsterdam, The Netherlands; 4 Department of Biomedical Physics and Biomedical Photonics, Amsterdam University Medical Center, University of Amsterdam, Amsterdam, The Netherlands; 5 Department of Neuroscience, Erasmus Medical Center, Rotterdam, The Netherlands; 6 Department of Ophthalmology, Radboud University Medical Center, Nijmegen, The Netherlands; 7 Institute of Molecular and Clinical Ophthalmology, Basel, Switzerland; 8 Department of Clinical Genetics, Erasmus Medical Center, Rotterdam, The Netherlands

**Keywords:** gap-junction delta-2, myopia, connexin 36, single nucleotide polymorphism, refractive error

## Abstract

Refractive errors are common eye disorders characterized by a mismatch between the focal power of the eye and its axial length. An increased axial length is a common cause of the refractive error myopia (nearsightedness). The substantial increase in myopia prevalence over the last decades has raised public health concerns because myopia can lead to severe ocular complications later in life. Genomewide association studies (GWAS) have made considerable contributions to the understanding of the genetic architecture of refractive errors. Among the hundreds of genetic variants identified, common variants near the gap junction delta-2 (*GJD2*) gene have consistently been reported as one of the top hits. *GJD2* encodes the connexin 36 (Cx36) protein, which forms gap junction channels and is highly expressed in the neural retina. In this review, we provide current evidence that links *GJD2*(Cx36) to the development of myopia. We summarize the gap junctional communication in the eye and the specific role of *GJD2*(Cx36) in retinal processing of visual signals. Finally, we discuss the pathways involving dopamine and gap junction phosphorylation and coupling as potential mechanisms that may explain the role of *GJD2*(Cx36) in refractive error development.

Myopia (nearsightedness) is the refractive error in which light focuses in front of the retina, resulting in blurred distant vision. This mismatch of the refractive power of the eyes is predominantly caused by an increase in ocular axial length. The prevalence of myopia has increased rapidly in the past few decades, up to 90% in East Asia and up to 42% in Europe at the age of 13 to 19 years.^1^ Although optical devices can correct myopic refractions, myopia can cause severe ocular complications, such as myopic macular degeneration, retinal detachment, and glaucoma.^2^ Particularly high myopia increases the risk of visual loss as one-third of those with severe myopic refractive errors develop irreversible visual impairment or blindness.^3^^,^^4^

Refractive errors, including myopia, originate from complex interactions between environmental and genetic risk factors. Low outdoor exposure levels and a high amount of near work are associated with myopia development.^5^ Genomewide association studies (GWAS) identified many common genetic variants for refractive error. In 2018, a meta-analysis of GWAS for refractive error included 160,420 participants and identified 161 independent genetic loci annotated to 233 candidate genes. Pathway analyses of these genomic hits suggested that, in particular, light processing in the retina is important for the development of refractive errors.^6^ The most recent meta-analysis of GWAS included even more participants (*n* = 542,934) and found an additional 336 loci. These findings elucidated the involvement of virtually all anatomic tissues of the eyes in refractive error development; and suggested a wide range of potential mechanisms (e.g. eye structure, ocular development, eye physiology, intraocular pressure, and circadian rhythm).^7^

The gap junction delta-2 (*GJD2*) gene is located in one of the first and most replicated myopia-associated loci found in independent study cohorts and ethnicities.^6^^–^^19^ SNPs near the *GJD2* gene have been associated with other myopia-related phenotypes, including ocular axial length, axial length/corneal radius ratio, and age of onset of myopia.^11^^,^^13^^,^^20^^,^^21^ Even though the identified single-nucleotide polymorphisms (SNPs) are not located inside the actual gene, *GJD2* is hypothesized to be the most biologically plausible gene in the locus.^15^^,^^18^

*GJD2* encodes connexin 36 (Cx36), a transmembrane protein that forms gap junction channels that play a role in intra- and intercellular communication by enabling the diffusion of ions and small molecules.^20^ Two different systems are in use for the nomenclature of this multigene family. *GJD2* is a combination of gap junction (GJ), its subclass based on sequence homology (D) and an Arabic numeral according to its order of discovery (2). Cx36 is based on its molecular weight predicted from the cDNA: approximately 36 kDa.^22^ Because both nomenclature systems are alternately used in the literature, we here refer to the gene/protein as a combination of both systems: *GJD2*(Cx36).

According to the human protein atlas, *GJD2*(Cx36) expression is enhanced (i.e. expressed at least 4 times the mean of other tissues) in the adrenal gland, pancreas, pituitary gland, and retina.^23^ *GJD2*(Cx36) containing gap junctions in the central nervous system facilitate electrical coupling between neurons and are present in various regions in the brain, predominantly in the inferior olive, olfactory bulb, and hippocampus.^24^^,^^25^ In the retina, *GJD2*(Cx36) containing gap junctions are present in photoreceptors (predominantly in cones), bipolar cells, amacrine cells, and ganglion cells.^26^^–^^32^

In the retina, *GJD2*(Cx36) plays an essential role in visual processing as it modulates signal-to-noise ratio by averaging noise through photoreceptor coupling and also contributes to night vision by transmitting rod-mediated visual signals.^33^^–^^36^ However, its role in emmetropization is still unresolved. Understanding the role of the myopia-associated SNPs found close to *GJD2*(Cx36), the function of *GJD2*(Cx36) and its role in visual processing is a starting point for disentangling the putative role of *GJD2*(Cx36) in refractive error development. In this review, we (1) summarize the genetic evidence for a role of *GJD2*(Cx36) in refractive error; (2) provide an overview about its structure, function, expression, and role in visual processing; (3) explore its conservation across species and discuss animal models which study *GJD2*(Cx36) in the context of myopia; (4) elaborate on the potential mechanisms by which *GJD2*(Cx36) might contribute to the pathogenesis of myopia; and (5) suggest future research directions.

## *GJD2*(Cx36) – Lessons Learned from Studies in Humans

In 2010, the first genetic locus identified in GWAS associated with refractive error was found at chromosome 15q14 (rs634990). This intergenic SNP is located 39 kb away from the 3′ end of *GJD2*(Cx36). Even though this SNP is also close to the *ACTC1* gene (74 kb from its 3′ end) and the *GOLGA8B* gene (180 kb from its 5′ end), *GJD2*(Cx36) was considered the most plausible candidate gene due to its expression in eye tissue (Table 1) and its role in the visual pathway (in the section: Expression of *GJD2*(Cx36) in the retina and the various functions in visual processing).^10^^,^^15^

**Table 1.** Expression of *GJD2*(Cx36) in Human Tissue

Database	Tissue	*GJD2* Expression Mean (std)	Unit	Based On (N)	Method
GTEx	Pituitary	10.51	TPM	283	RNA seq
	Brain-frontal cortex	2.75		209	
IOWA	Retina	49.11	PLIER score	6	RNA expression chip
	Trabecular meshwork	44.15		6	
	Ciliary body	41.50		6	
	Optic nerve	39.05		6	
	Choroid RPE	32.54		6	
	Sclera	31.18		6	
	Lens	24.77		6	
	Iris	22.60		6	
	Optic nerve head	20.99		6	
	Cornea	11.54		6	
Fantom5	Pituitary	33.20	Scaled tags per million	1	RNA seq
	Retina	12.50		Mixed	
HPA Atlas	Adrenal gland	9.90	pTPM	3	RNA seq
	Cerebral cortex	2.40		3	
	Pancreas	1.00		2	
Booij et al. 2009^42^	RPE	30.00 (9.30)	percentiles	6	RNA expression chip
	Photoreceptors	29.60 (3.40)		6	
	Choroid	35.80 (10.30)		6	
Young et al. 2013^43^	Adults optic nerve	−0.57	Avg signal	6	RNA expression chip
	Fetal optic nerve	−2.05		15	
	Adult cornea	−0.82		6	
	Fetal cornea	−0.40		15	
	Adult retina	8.42		6	
	24 week retina/RPE	11.10		15	
	12 week Ret/ RPE/Chr	13.84		15	
Li et al. 2014^44^	Macular retina	22.54	FPKM	8	RNA expression chip
	Macular retinal pigment epithelium/choroid/sclera	0.00		8	
	Peripheral retina	22.17		8	
	Peripheral RPE/Chr/sclera	0.00		8	
Cowan et al. 2020^45^	Rods	per = 0.00513%, fov = 0.00353%	NTP	3	single cell RNA seq
	Cones	per = 0.02973%, fov = 0.01649%		3	
	Horizontal cells	per = 0.00047%, fov = 0.00020%		3	
	On BCs	per = 0.00165%, fov = 0.00219%		3	
	OFF BCs	per = 0.00321%, fov = 0.00139%		3	
	Acs	per = 0.01546%, fov = 0.02133%		3	
	GCs	per = 0.00094%, fov = 0.00023%		3	
	Glycinergic Acs	per = 0.00046%, fov = 0.00014%		3	
	RPE	per = 0.00010%, fov = 0.00005%		3	

RNA expression data from expression chips (IOWA, Bergen et al., Young et al., and Stambolian et al.) and RNAseq data (GTEx, Fantom5, HPA atlas, and Cowan et al.). Data from Bergen AAB et al., is shown in percentiles. In Young TL et al., a strong signal is defined as >40. These data include microarray data from gene expression chips. Data from Stambolian DE et al., presents fragments per kilobase of gene per million mapped reads. In Cowan et al., gene expression is shown as a percentage of normalized transcripts.

Abbreviations: GTEx, Genotype-Tissue Expression; IOWA, the ocular tissue database; accessed via https://genome.uiowa.edu/otdb/; HPA, Human Protein Atlas; TPM, transcripts per million; pTPM, protein-coding transcripts per million; Avg signal, average values for each tissue type from raw, un-normalized data; FPKM, fragments per kilobase of gene per million mapped reads; NTP, normalized transcript percentages; Ret, retina; RPE, retinal pigment epithelium; Chr, choroid; BCs, bipolar cells; Acs, amacrine cells; GCs, ganglion cells; per, peripheral; fov, foveal.

After this first finding, another SNP in the same locus was identified; rs524952 (minor allele frequency [MAF] 0.46).^17^ Both rs634990 and rs524952 are in high linkage disequilibrium (r^2^ and D′ = 1) and have been consistently replicated in multiple GWAS of refractive error (Table 2, Supplementary Table S1).^6^^,^^7^^,^^9^^–^^11^^,^^13^^,^^15^^–^^19^^,^^21^^,^^37^^,^^38^ Rs524952 was reported as the most significant SNP in the latest meta-analysis of refractive error GWAS.^7^ Until now, all SNPs identified at the 15q14 locus are intergenic, whereas coding variants in the *GJD2*(Cx36) gene itself have not been associated with refractive error. This suggests that regulatory variants rather than coding variants in *GJD2*(Cx36) play a role.^15^ This notion is further supported by data from the Genotype-Tissue Expression (GTEx) database, a public resource that examines human tissue-specific gene expression and regulation, which reports that both rs634990 and rs524952 influence the expression of *GJD2*(Cx36) in the pancreas and pituitary.^39^ The minor alleles rs634990_C and rs524952_A are associated with lower expression levels of *GJD2*(Cx36) in these tissues. Following this line of thought, one can hypothesize that downregulation of *GJD2*(Cx36) leads to an increased risk of myopia. However, it is worth noting that the GTEx database does not include eye tissue, therefore, limiting the interpretation of the results described in pancreas and pituitary.

**Table 2.** Summary of the Studies and Study Design in Which rs634990 and rs524952 Have Been Associated With Myopia or Related Phenotypes

Variant	Pos (hg38)	Ref	Alt	Discovery Study	Study Design	Outcome	Cohort (N)	Replicated in	Study Design	Trait	Study Cohorts (N)
rs634990	34713872	T	C	Solouki *et al*. (2010)^15^	GWAS	MSE	RS-I (5,328) and RS-2 and 3, Erasmus Ruchpen Family Study an Twins UK (replication, 10,280).	Stambolian et al. (2013)^16^	GWAS meta-analysis	MSE	AREDS; KORA; FES; OGP-Talana, the Multiethnic Study of Atherosclerosis (7,280 [26,953 replication])
								Hysi et al. (2010)^10^	GWAS	Refractive error	TwinUK (4270)
								Verhoeven et al. (2012)^17^	GWAS meta-analysis	MSE	31 studies from CREAM (49363)
								Schache et al. (2013)^13^	Genetic association study	Refractive error	BMES (1571)
								Schache et al. (2013)^13^	GWAS	Axial length	BMES (1571)
rs524952	34713685	T	A	Verhoeven et al. (2012)^17^	GWAS meta-analysis	MSE	31 studies from CREAM (49363)	Stambolian et al. (2013)^16^	GWAS meta-analysis	MSE	AREDS; KORA; FES; OGP-Talana, the Multiethnic Study of Atherosclerosis (7,280 [26,953 replication])
								Schache et al. (2013)^13^	Genetic association study	Refractive error	BMES (1571)
								Simpson et al. (2013)^38^	GWAS	Refractive error	AREDS (2000)
								Verhoeven et al. (2013)^18^	GWAS meta-analysis	MSE	32 studies from CREAM (45,758)
								Kiefer et al. (2013)^11^	Survival analysis	Age of first spectacle wear (23 and me)	23andMe (45,771)
								Hayashi et al. (2011)^9^	Case-control design	High myopia	Japanese (1125 vs. 366 [cataract] or 929 [healthy])
								Tideman et al. (2016)^21^	Meta-analysis of linear regression	AL/CR ratio	18 cohorts from CREAM (26,764)
								Yoshikawa et al. (2014)^19^	GWAS	MSE	The Nagahama Study (3712)
								Fan et al. (2016)^37^	GxE: meta-analysis of linear regression MSE and educational attainment	MSE	34 studies from CREAM (50,351)
								Tedja et al. (2018)^6^	GWAS	MSE	37 studies from CREAM and two from 23andMe (discovery 160,420 and replication 95,505)
								Hysi et al. (2020)^7^	GWAS meta-analysis	MSE	UK Biobank, GERA, 23andMe, and CREAM Consortium studies (542,934)

Abbreviations: RS, Rotterdam Study; MSE, mean spherical equivalent; AREDS, Age-Related Eye Disease Study; KORA, Cooperative Health Research in the Region of Augsburg; FES, Framingham Eye Study; OGP-Talana, the Ogliastra Genetic Park-Talana; CREAM, Consortium for refractive error and myopia; BMES, Blue Mountains Eye Study; AL/CR, axial length corneal radius ratio; GERA, Genetic Epidemiology Research on Adult Health and Aging.

To further explore the potential regulatory role of the SNPs identified at 15q14, we examined whether variants in moderate linkage disequilibrium (LD; R^2^ > 0.2) with the first associated SNP, rs634990, overlapped with regulatory elements of the ENCODE data. Moreover, we retrieved their RegulomeDB score, which is a score that assesses the evidence of a SNP for regulatory potential. We assessed a total of 102 SNPs (see Supplementary Table S1) of which 15 have been identified in refractive error GWAS and 14 of them were replicated in at least one other study.^6^^,^^7^^,^^9^^–^^11^^,^^13^^–^^21^^,^^37^^,^^38^^,^^40^ Twelve SNPs out of 102 showed moderate evidence of a location in a regulatory region (Table 3). These 12 SNPs have a RegulomeDB score of 3a, which provides evidence for a localization of the SNP in a transcription factor binding site, in any motif, or a DNase peak; none of the SNPs showed high evidence to be a regulatory variant (i.e. RegulomeDB score 1a–f and 2a–c). In total, 44% (45/102) of the SNPs overlapped with at least three regulatory elements of the ENCODE database (i.e. promoter or enhancer histone marks, DNase I hypersensitive sites, transcription factor, or other protein-binding sites, and expression quantitative trait loci [eQTLs]; see Supplementary Table S1). This finding supports the hypothesis that SNPs associated with refractive error at the *GJD2*(Cx36) locus may influence the phenotype through gene regulation. However, as described for the GTEx database, the ENCODE data does not include eye tissues or retinal cells, therefore, we are cautious in drawing strong conclusions from this dataset.

**Table 3.** Variants in LD With rs634990

SNPs in LD With rs634990 Showing Evidence to be Regulatory Variants							HaploReg. Version 4.1 Annotation											
Variant	Identified in (PMID)	Pos (hg38)	Ref	Alt	LD (r2, in Relation to rs634-990)	LD (D', in Relation to rs634-990)	GERP Cons	Prom-oter Histone Marks (Roa-dmap)	Enha-ncer Histone Marks (Road-map)	DNA se	Proteins Bound	Motifs Changed	eQTL Results	Ref Seq Genes	dbSNP Functional Annotation	Query SNP Overlaps With ENC-ODE Data (≥ 2 Elem-ents)±	Query SNP overlaps with ENC-ODE Data (≥ 3 Elem-ents)±	Regu-lome DB v. Regu-lome DB_Score
rs634990	20835239, 23474815, 20835236, 22665138, 23131718	34713872	T	C	NA	NA	yes		HRT	KID		6 altered motifs	2 hits	39kb 3' of GJD2	**intergenic**	yes	yes	**5**
rs524952	22665138, 23474815, 23131718, 24227913, 23396134, 23468642, 21436269, 27611182, 25335978, 27020472, 29808027, 32231278	34713685	T	A	1	1			HRT			AFP1,SIX5	2 hits	39kb 3' of GJD2	**intergenic**	yes	yes	**7**
rs685352	22665138, 23474815, 23131718, 24227913	34716134	A	G	0.86	0.99			LIV, PANC, MUS			5 altered motifs	2 hits	36kb 3' of GJD2	**intergenic**	yes	yes	**3a**
rs688220	22665138, 23474815, 23131718, 24227913	34706674	G	A	0.6	0.84				BLD,BRN			2 hits	46kb 3' of GJD2	**intergenic**	yes	no	**5**
rs560766	22665138, 23474815, 23131718, 24227913	34708741	G	A	0.6	0.84		BRN, HRT, PANC	7 tissues	15 tissues	CTCF,GATA1	Msx-1	2 hits	44kb 3' of GJD2	**intergenic**	yes	yes	**4**
rs619788	22665138, 23474815, 23131718, 24227913	34702905	C	A	0.58	0.83	yes					HNF1	2 hits	50kb 3' of GJD2	**intergenic**	yes	no	**7**
rs580839	22665138, 23474815, 23131718, 24227913	34706628	G	A	0.58	0.84				BLD,BRN		CACD,GR	2 hits	46kb 3' of GJD2	**intergenic**	yes	yes	**5**
rs9920099		34701123	C	T	0.57	0.83			5 tissues	5 tissues	CEBPB	4 altered motifs	1 hit	51kb 3' of GJD2	**intergenic**	yes	yes	**3a**
rs7176510	22665138, 23474815, 23131718, 24227913	34707278	C	T	0.57	0.83						20 altered motifs	1 hit	45kb 3' of GJD2	**intergenic**	yes	no	**5**
rs4924134	22665138, 23474815, 23131718, 24227913	34702364	A	G	0.56	0.83			BRN			8 altered motifs	1 hit	50kb 3' of GJD2	**intergenic**	yes	yes	**5**
rs11073058	22665138, 23474815, 23131718, 24227913, 24144296, 25823570	34697425	G	T	0.54	0.81			ESC, IPSC, BLD			CHD2	1 hit	55kb 3' of GJD2	**intergenic**	yes	yes	**7**
rs11073059	22665138, 23474815, 23131718, 24227913	34697473	T	A	0.54	0.81			ESC, IPSC, BLD			GR	1 hit	55kb 3' of GJD2	**intergenic**	yes	yes	**7**
rs11073060	22665138, 23474815, 23131718, 24227913	34697650	C	A	0.54	0.81			IPSC, BLD			10 altered motifs	1 hit	55kb 3' of GJD2	**intergenic**	yes	yes	**6**
rs7163001	22665138, 23474815, 23131718, 24227913	34698373	G	A	0.54	0.82						Arid5b, HDAC2, Nanog	1 hit	54kb 3' of GJD2	**intergenic**	yes	no	**5**
rs678510		34711108	T	C	0.51	0.99			ESDR			CCNT2, GATA, TATA	2 hits	41kb 3' of GJD2	**intergenic**	yes	yes	**3a**
rs652158		34719259	A	G	0.49	0.79			10 tissues	HRT, KID, CRVX		4 altered motifs	2 hits	33kb 3' of GJD2	**intergenic**	yes	yes	**3a**
rs684374		34716391	G	C	0.47	0.99		MUS	6 tissues	6 tissues	GR	GR, Myf, TCF12	2 hits	36kb 3' of GJD2	**intergenic**	yes	yes	**3a**
rs513587		34709645	T	C	0.46	0.99			8 tissues			10 altered motifs	1 hit	43kb 3' of GJD2	**intergenic**	yes	yes	**3a**
rs8032019	22665138, 23474815, 23131718, 24227913	34699289	A	G	0.44	0.85						13 altered motifs		53kb 3' of GJD2	**intergenic**	no	no	**3b**
rs3932344		34708040	T	C	0.44	0.86						6 altered motifs		44kb 3' of GJD2	**intergenic**	no	no	**3a**
rs4924135		34702599	A	C	0.43	0.85						4 altered motifs		50kb 3' of GJD2	**intergenic**	no	no	**3a**
rs6495707		34691197	G	A	0.34	0.82			6 tissues	11 tissues	STAT3	Smad3, VDR		61kb 3' of GJD2	**intergenic**	yes	yes	**3a**
rs56062557		34692394	T	G	0.34	0.82			BLD			GR, NF-kappaB		60kb 3' of GJD2	**intergenic**	yes	no	**3a**
rs17237002		34692429	C	G	0.34	0.82			BLD			Nanog, Sox		60kb 3' of GJD2	**intergenic**	yes	no	**3a**
rs1370156	25233373	34692682	G	C	0.21	0.67			BLD			Pax-5	1 hit	60kb 3' of GJD2	**intergenic**	yes	yes	**5**
rs649782		34712867	A	C	0.21	0.63						10 altered motifs		40kb 3' of GJD2	**intergenic**	no	no	**3a**

SNPs in LD with rs634990 showing evidence to be regulatory variants or which were identified in certain study. Using the software HaploReg (version 4.1) (Ward and Kellis 2012) and RegulomeDB version 1.1 (Boyle et al. 2012), we investigated regulatory annotations for variants in LD (r2 > 0.2, 1000 genomes CEU) with the refractive error SNPs annotated to GJD2(Cx36). Using HaploReg version 4.1 all variants were extracted and examined for overlap with regulatory elements of the ENCODE data. RegulomeDB score was used to assess their potential functional consequence, as described previously (Schaub et al. 2012).

Besides refractive error, the 15q14 locus, including the *GJD2*(Cx36) gene, has been associated with other myopia-related proxies, including axial length and “age of first spectacle wear.” The Blue Mountain Eye study reported an association with axial length.^13^ Subsequently, *GJD2*(Cx36) was replicated in a GWAS of axial length, including both European and Asian populations^20^ and in a Japanese study.^40^ Refractive error GWAS generally use spherical equivalent as the outcome, a calculated value in which the spherical value and half the cylindrical value are summed. Both spherical equivalent and ocular axial length are highly correlated, explaining the shared genetic association with the 15q14 locus for these traits.^41^ In another study, *GJD2*(Cx36) was identified using the survival analysis parameter “age of first spectacle wear” as a proxy for myopia.^11^ Because a younger age of onset generally leads to higher degrees of myopia, it is not surprising that *GJD2*(Cx36) was also identified in this study.^11^^,^^46^^–^^49^

Contrary to several other candidate genes associated with refractive error, mutations in *GJD2*(Cx36) have not been reported to cause a human Mendelian disorder. One could speculate that *GJD2*(Cx36) is either a crucial gene for embryogenesis, or, on the other side of the spectrum, a gene tolerant to genetic variation (low constraint). Genes involved in dominant Mendelian disorders are known to be highly intolerant to variation (high constraint). Databases, such as gnomAD, facilitate the interpretation of variants and indicate how intolerant a gene is to variation by providing constraint metrics.^50^ These metrics include the probability of loss-of-function intolerance (pLI) and the loss-of-function o/e upper bound fraction (LOEUF). A pLI of 1 and a LOEUF <0.35 have been widely used as a threshold to indicate high intolerance to variation. According to gnomAD, *GJD2*(Cx36) shows a pLI = 2 and a LOEUF = 0.7, this indicates that *GJD2*(Cx36) is moderately tolerant.

## *GJD2*(Cx36)

### Physical Structure of the *GJD2*(Cx36)

**Figure 1.**
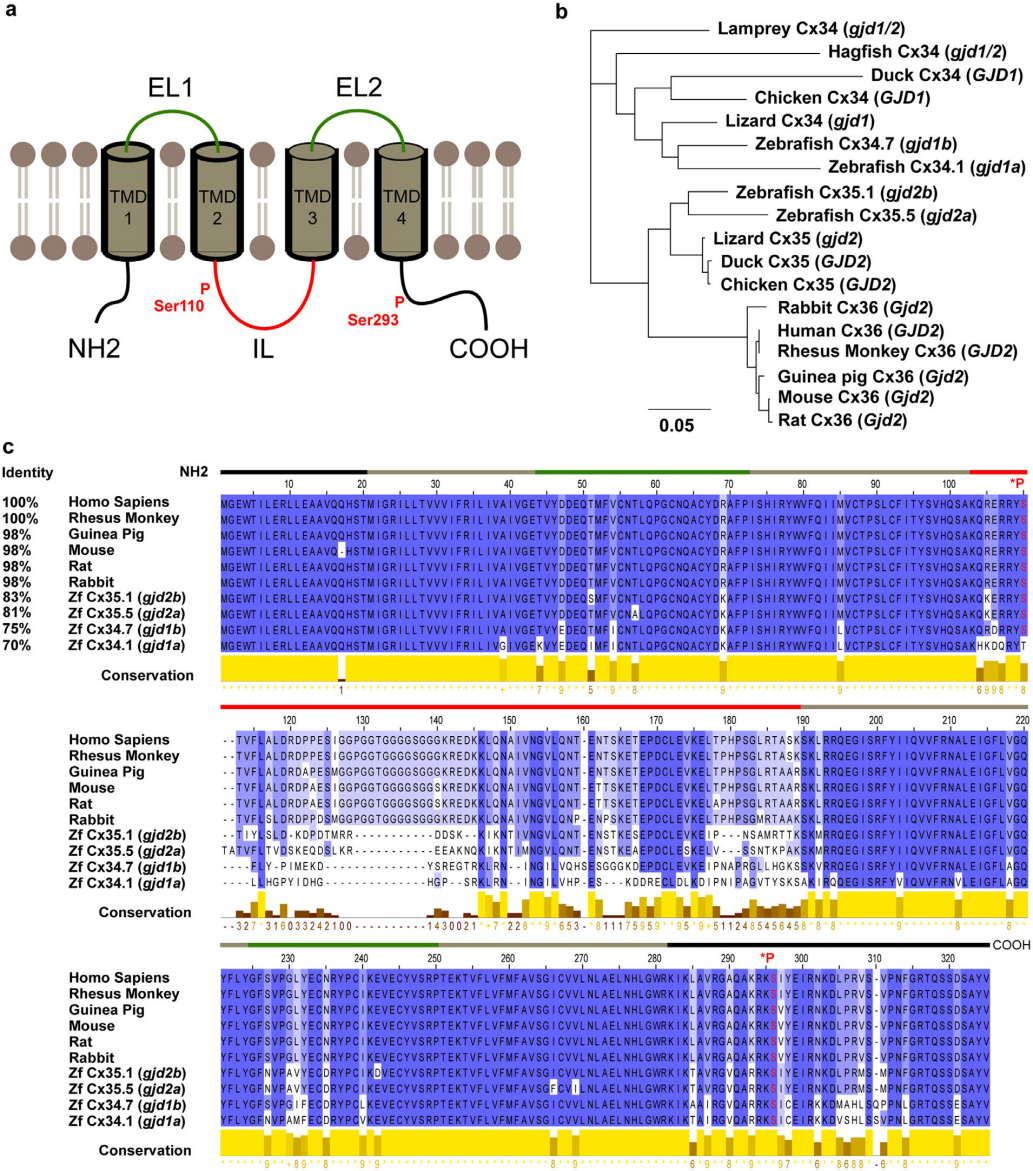
**Structure and conservation of *GJD2*(Cx36)**. Panel (**A**) shows the *GJD2*(Cx36) protein consisting of four transmembrane domains (TMD1-4), alternated by two extracellular loops (EL1-2), with the phosphorylation sites on the intracellular loop (IL; Ser110) and on the C-terminus (COOH; Ser293, Ser276 for zebrafish). Panel (**B**) shows the conservation of *GJD2*(Cx36) throughout commonly used species for myopia research. The phylogenetic tree in vertebrate lineages of reptiles and birds shows two subfamilies, *GJD2*(Cx35/Cx36) and *GJD1*(Cx34). The latter paralog is not present in mammals, whereas in teleost fish four functional orthologs have been identified (gjd1a(Cx34.1), gjd1b(Cx34.7), gjd2b(Cx35.1), and gjd2a(Cx35.5)). Panel (**C**) shows that for mammalian species 98% to 100% and for zebrafish 70% to 83% of the *GJD2*(Cx35/Cx36) protein is conserved relative to the human protein. Intergenic variation is mainly located in the intracellular loop (red trace) and in the C-terminus (final black trace), whereas the two phosphorylation sites are conserved throughout all species *P).

Cx36 is a membrane protein containing one cytoplasmic N-terminus, four transmembrane helices, two extracellular loops, one cytoplasmic loop, and one C-terminal tail (Fig. 1A). To date, 21 genes in the human genome have been identified to encode distinct but structurally related isoforms of gap junction proteins.^51^

Six gap junction proteins assemble into hexameric channels, called connexons or hemichannels. At the plasma membrane, two hemichannels from two adjacent cells connect and form a gap junction. Distinct gap junction proteins can be co-expressed in the same cell. If the hemichannel consists of only one subtype of gap junction protein (e.g. 6 *GJD2*(Cx36)), it is called a homomeric hemichannel, contrary to a heteromeric hemichannel, which contains different gap junction proteins. Similarly, if two adjacent cells have a distinct composition of the hemichannels, then the cell-to-cell gap junction channel is referred to as heterotypic. An example of this is the *GJD2*(Cx36)-containing hemichannel in an AII amacrine cell together with *GJA7*(Cx45)-containing hemichannel in an ON cone bipolar cell.^52^ Moreover, gap junctional plaques, containing multiple gap junction channels, can be composed of a random mixture of homomeric channels, heteromeric channels, or a combination of both.^51^

### Functions of Gap Junction Proteins

Functions of the gap junction proteins can be divided into three categories.^53^ The first and most well-known is gap junction intercellular communication, which can be either ionic or biochemical. Ionic communication refers to the passive diffusion of cytoplasmic (cat)ions (e.g. Na+, K+, and Ca2+), contributing to essential functions ranging from the contraction of cardiac myocytes to the propagation of action potentials via electrical synapses.^54^ In contrast, the exchange of small molecules and metabolites (e.g. cAMP) up to 1000 Dalton in size, referred to as biochemical transport, play a role in cellular homeostatic processes.^55^

Second, gap junction proteins perform essential roles as hemichannels. After oligomerization of the six gap junction proteins, hemichannels are transported to and inserted into the plasma membrane, where they may remain uncoupled. They are involved in various functions during cell life (i.e. proliferation, development, survival, and death), controlled all by both intracellular and extracellular factors.^56^^,^^57^ In the retina, feedback from horizontal cells to photoreceptors depends strongly on gap junction hemichannels.^58^^,^^59^ There is evidence that *GJD2*(Cx36) forms functional hemichannels in the pancreas and neuronal cell cultures. However, it is unclear whether *GJD2*(Cx36) hemichannels play a role in visual processing in the retina.^60^^,^^61^

Third, various studies have shown that connexins can function independent of their gap junction- and hemichannel-forming properties. Although their mechanistic aspects remain largely unknown, recent findings suggest that connexins interact with other proteins, including tight junction proteins, ZO-1, occludin, claudins, N-cadherin, and the cytoskeletons, microtubules, actin, and catenins.^62^^–^^66^ In this way, they are capable to modulate gene expression indirectly by inducing secondary effects.^67^^–^^70^

It is worth noting that modulation of gap junction permeability is essential for normal physiological processes. Gap junction permeability is determined by multiple factors, including channel composition (i.e. heterotypic versus heteromeric), modulation of gap junction protein expression and post-translational modifications (i.e. phosphorylation).^22^^,^^71^^–^^73^ Phosphorylation of *GJD2*(Cx36) is further discussed in the section: Regulation of expression and phosphorylation of *GJD2*(Cx36).

### Expression of *GJD2*(Cx36) in the Retina and the Various Functions in Visual Processing

*GJD2*(Cx36) is expressed in a number of retinal cell types and plays a role in signal transmission in the retina. Here, we discuss the current evidence of expression of connexins per cell type (see Table 1) and the specific roles of *GJD2*(Cx36) in visual processing.

Before elaborating on the role of *GJD2*(Cx36), we first summarize the successive steps in visual processing. Light is converted into a neuronal signal by photoreceptors, which can be classified into two types; rods for scotopic vision and cones for photopic vision. The photoreceptor synapses onto bipolar cells, which are interneurons classified based upon the source of the signal: rod bipolar cells receive input from rods and cone bipolar cells from cones. Cone bipolar cells can be distinguished in ON and OFF types. ON bipolar cells depolarize upon increasing light stimulation, whereas OFF bipolar cells respond with a hyperpolarization and vice versa. Subsequently, the signal is transmitted from cone bipolar cells to the retinal ganglion cells. In addition, horizontal cells and amacrine cells provide lateral connections between neurons (e.g. connecting one bipolar cell to another bipolar cell), modulating the signal. Rod bipolar cells do not synapse directly to ganglion cells. Instead, they synapse to AII amacrine cells, which in turn signal to ganglion cells and cone bipolar cells (primary rod pathway). The signal exits the retina via the axons of the ganglion cells, the retinal nerve fiber layer, and the optic nerve for further processing in the brain.

**Figure 2.**
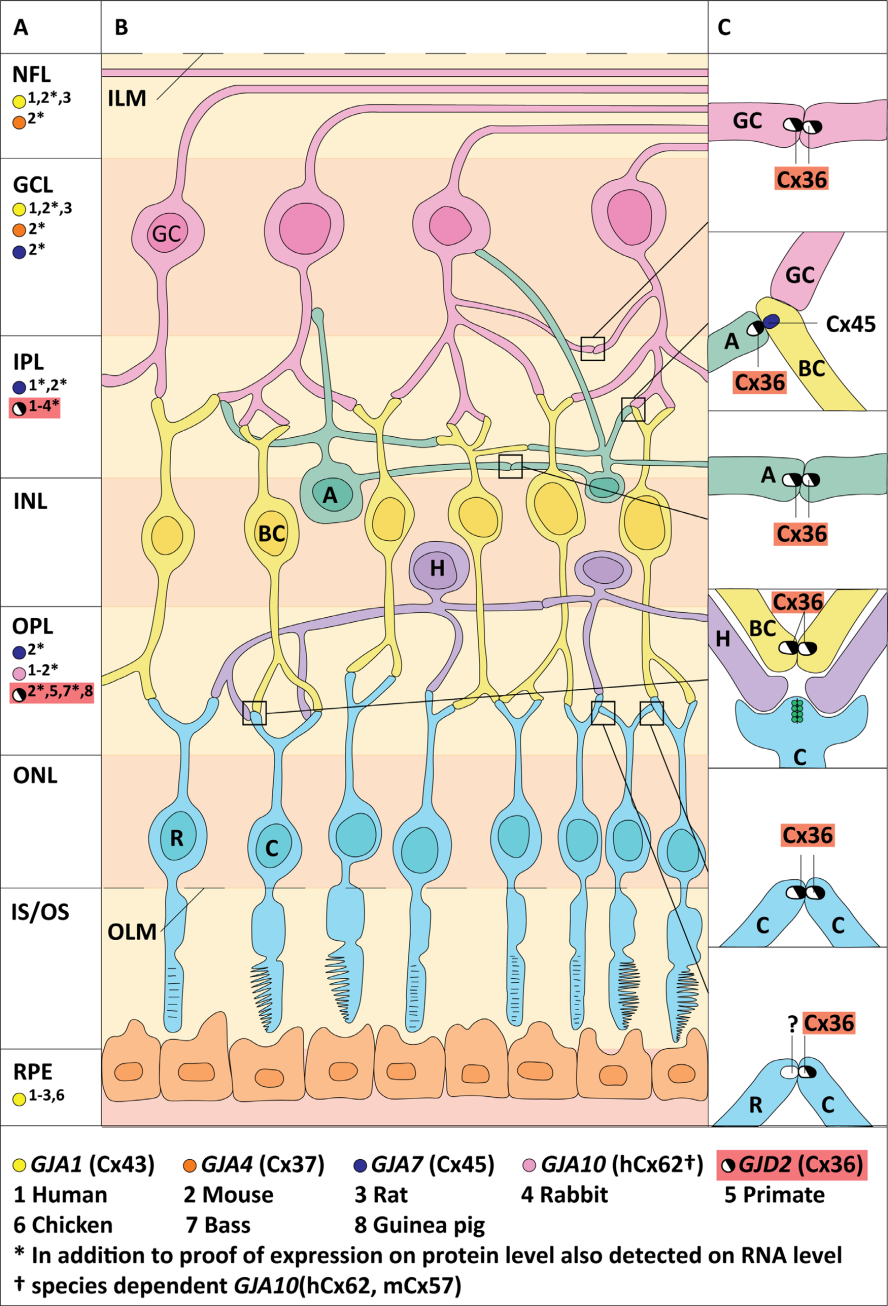
**Gap junction proteins in the human retina and their coupling with *GJD2*(Cx36).** Panel (**A**) shows the gap junction proteins present in the different retinal layers. Panels (**B**) and (**C**) show the gap junction coupling that contains *GJD2*(Cx36). Dot colors define the different gap junction proteins. Numbers represent the species for which the gap junction locations have been described. Gap junctions are detected at the protein level, when additionally detected on RNA (including cDNA) level, color dots are marked with an asterisk. Homotypic GJD2(Cx36)-containing gap junctions are present between dendrites of alpha ganglion cells, between dendrites of AII amacrine cells, between (ON/OFF) cone bipolar cells, between cones, and between rods and cones. For the latter homotypic configuration, most evidence localizes *GJD2*(Cx36) on the cone side. *GJA7*(Cx45) is the only gap junction protein forming channels with *GJD2*(Cx36). Heterotypic channels are present between subtypes of amacrine cells (providing *GJD2*(Cx36)) and (ON/OFF) cone bipolar cells (providing *GJA7*(Cx45)). *GJA1*(Cx43), *GJA4*(Cx37), and *GJA10* (hCx62, mCx57, and pCx60) are the remaining gap junction proteins in the retina, localized between horizontal cells, but these do not colocalize with *GJD2*(Cx36). In addition to the gap junctions visualized in the figure, *GJD2*(Cx36) gap junctions have been reported between amacrine cells and alpha ganglion cells. Abbreviations: A, amacrine cell; BC, bipolar cells; C, cone photoreceptor cell; GC, ganglion cell; GCL, ganglion cell layer; H, horizontal cell; ILM, inner limiting membrane; INL, inner nuclear layer; IPL, inner plexiform layer; IS, inner segment; NFL, optic nerve fiber layer; OLM, outer limiting membrane; ONL, outer nuclear layer; OPL, outer plexiform layer; OS, outer segment; R, rod photoreceptor cell; RPE, retinal pigmented epithelium.

Figure 2 provides an overview of the current evidence of the expression of *GJD2*(Cx36) and other gap junction proteins in the retina (see Fig. 2A) and the localization of homo- and heterotypic *GJD2*(Cx36)-containing gap junctions between various cell types in the retina (see Figs. 2B, 2C).

#### *GJD2* in Photoreceptors and Bipolar Cells

*GJD2*(Cx36) is present between cones and between rods and cones, predominantly identified on the cone side (see Fig. 2).^27^^,^^30^^,^^74^^–^^76^ However, one study reported *GJD2*(Cx36) expression in rod photoreceptors.^26^ In addition, *Gjd2*(Cx36)-containing gap junctions are located between the dendrites of (ON/OFF) cone bipolar cells, close to the cone pedicles.^27^^,^^74^^,^^77^ This finding has been confirmed in human tissue (see Table 1).^32^^,^^42^^–^^45^^,^^78^ *GJD2*(Cx36)-containing gap junctions between cones are considered to improve signal-to-noise-ratio by averaging out noise generated by sources intrinsic to the photoreceptors, whereas the signal evoked by a uniform stimulus is not affected.^34^^–^^36^

#### *GJD2* in Ganglion Cells and Amacrine Cells

*GJD2*(Cx36) forms homotypic dendrodendritic gap junctions between alpha ganglion cells and between AII amacrine cells (see Fig. 2).^31^^,^^79^^–^^81^ Additionally, alpha ganglion cells and AII amacrine cells are also connected to each other by *GJD2*(Cx36) gap junctions^31^^,^^82^ (not depicted in Fig. 2).

AII amacrine cells are the central nodes in the primary rod pathway. They relay the input received from rod bipolar cells to ON cone bipolar cells via *GJD2*(Cx36) gap junctions and to OFF cone bipolar cells via glycinergic inhibition. The secondary rod pathway, which depends on *GJD2*(Cx36) gap junctions between rods and cones, also relays rod signals to the cone pathway.^26^^,^^27^^,^^33^^,^^35^^,^^37^ The known rod pathways differ in light sensitivity, with the primary rod pathway being the most sensitive, followed by the secondary rod pathway.^26^^,^^85^ As such, *GJD2*(CX36) has an important role in rod signaling under dim light conditions. In line with this, scotopic electroretinograms (ERGs) of *Gjd2*(Cx36) knockout mice showed a reduction of the b-wave, which represents the ON cone bipolar response.^26^^,^^86^^,^^87^ The presence of a residual b-wave indicates that night vision's ON component is not entirely dependent on *GJD2*(Cx36), as was further substantiated by the finding that optokinetic reflexes could still be elicited in Cx36 knockout mice.^88^

*GJD2*(Cx36) dependent rod-pathways also contribute to dopamine release from dopaminergic amacrine cells (DACs) through excitatory ON cone bipolar cell input.^89^ DACs provide negative feedback via inhibitory projections to ON cone bipolar cells^90^ and synapse onto AII as well as A17 amacrine cells in the rod pathway. Light-evoked responses of DACs are modulated by inhibitory synaptic input from glycinergic and GABAergic amacrine cells,^91^^,^^92^ which are driven by OFF cone bipolar cells that receive glycinergic inhibition from AII amacrine cells. Although DACs receive excitatory ON inputs from all photoreceptor types, the *GJD2*(Cx36) dependent rod-pathway dominates the input during dim light conditions.^89^ Underscoring the tight convolution of the rod pathways with the dopaminergic system, rod pathway deficiency negatively affects DAC numbers and retinal dopamine/DOPAC levels, as well as the myopic shift in response to form deprivation in mice.^93^^–^^96^

The rod pathway and dopaminergic system are closely involved in the regulation of circadian clocks in the eyes, which have a probable role in myopia development.^97^^–^^101^ Remarkably, rod photoreceptors can drive circadian photoentrainment across a wide range of light intensities.^102^ *GJD2*(Cx36) dependent rod pathways play a particular role in entrainment of the retinal circadian clock, enabling the induction of phase-shifts of the retinal clock by short-duration light pulses in the visible part of the spectrum.^103^

#### Type of *GJD2*(Cx36) Gap Junction Connections

In the retina, *GJD2*(Cx36) forms homotypic as well as heterotypic gap junctions, the latter exclusively with *GJA7*(Cx45). These heterotypic gap junctions are formed by amacrine cells expressing *GJD2*(Cx36) and by ON cone bipolar cells expressing *GJA7*(Cx45) (see Fig. 2C).^28^^,^^31^^,^^82^^,^^87^^,^^104^^–^^106^ Besides AII amacrine cells, also subtype A8 amacrine cells appear to be connected through heteromeric junctions.^107^ Although some reports contradict a heterotypic connection between amacrine and bipolar cells, the general notion is that the type of connection depends on the specific function of the bipolar subtype.^26^^,^^87^^,^^105^

### Other Connexins in the Eyes

Aside from *GJD2*(Cx36), many other connexins are present in the retina. Apart from its ability to form heterotypic connection, *GJA7*(Cx45) also forms homotypic gap junctions between ganglion cells.^86^^,^^108^ *GJA10*(Cx62, mCx57), *GJA1*(Cx43), and *GJA4*(Cx37) are other connexins expressed in the retina.^32^^,^^86^^,^^109^^–^^113^ *GJA10*(hCx62) has been detected in the human retina and its mouse homolog, *Gja10*(mCx57), has been exclusively localized in homomeric gap junctions between horizontal cells in adult mice (see Fig. 2).^32^^,^^111^^,^^114^^,^^115^ *GJA1*(Cx43) may occur around Muller glia in the nerve fiber layer, partly around blood vessels in the ganglion cell layer and as hemichannels in the retinal pigmented epithelium.^49^^,^^86^^,^^116^^–^^118^ *GJA4*(Cx37) has been identified in endothelial cells of blood vessels in the ganglion cell layer.^86^

**Figure 3.**
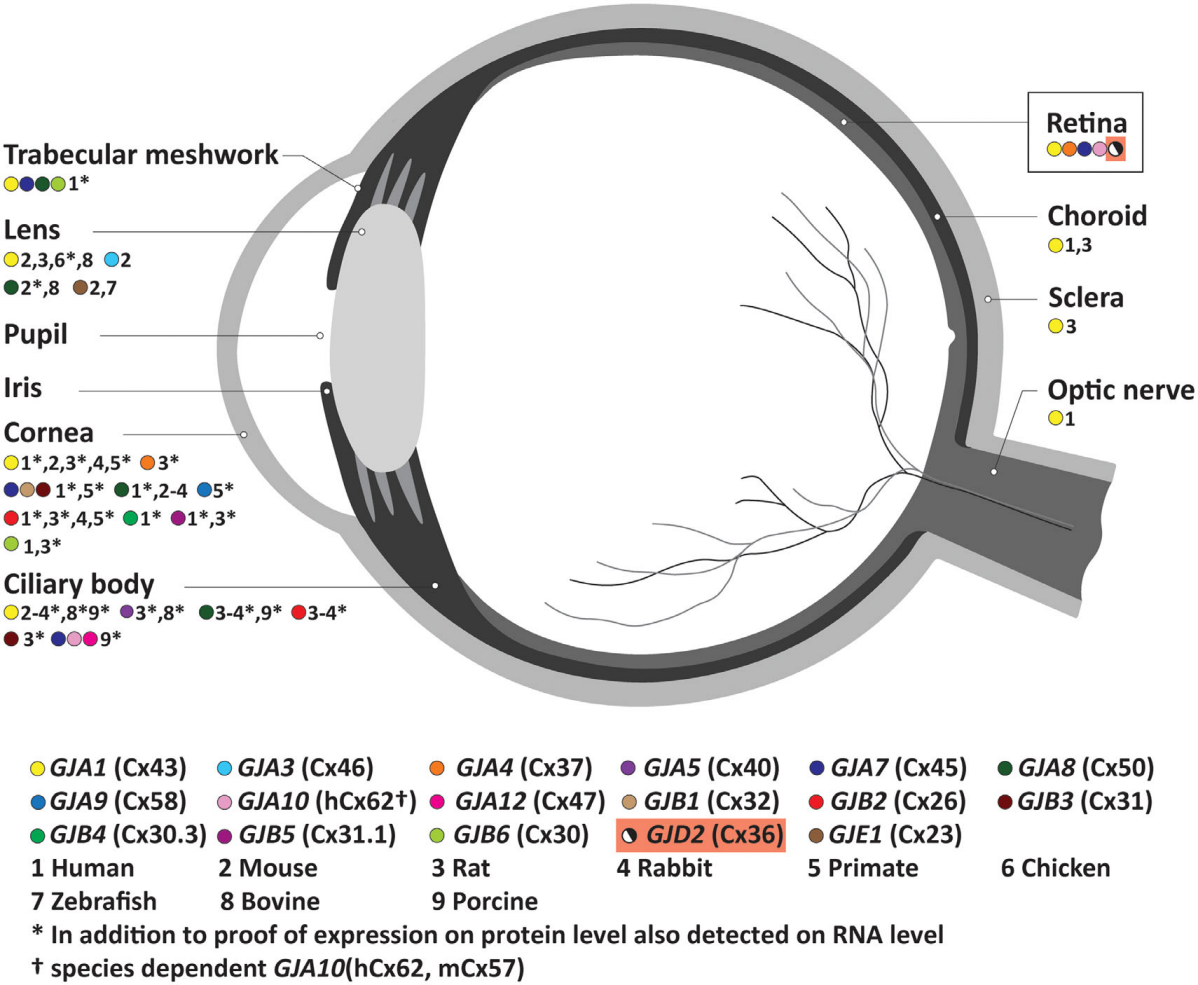
**Gap junction proteins (connexins) in the eye.** Color dots define the different gap junction proteins. Numbers represent the species for which the gap junction locations have been described. Gap junctions are detected at the protein level, when additionally detected on RNA (including cDNA) level color dots are marked with an asterisk. *GJD2*(Cx36) is expressed in the retina exclusively. *GJA1*(Cx43) is widely expressed throughout the eyes and is identified in all ocular segments.

Even more gap junction proteins than mentioned above are present in other parts of the eyes (Fig. 3). Of all the connexins, *GJA1*(Cx43) is most widely expressed in all ocular components. In the cornea of humans and other species, at least nine gap junction proteins are expressed, mediating intercellular communication to maintain corneal homeostasis.^119^^,^^120^ Within both the ciliary body and trabecular meshwork a variety of gap junction proteins are identified, which seem to be essential for the regulation of the intraocular pressure.^121^^–^^123^ Gap junction proteins in the lens are required for its transparency, as has been demonstrated in multiple species.^124^^–^^132^ Although *Gja8*(Cx50) has been implicated in ocular growth, no evidence points toward a potential contribution to refractive error.^133^ Interestingly, *GJA1*(Cx43) is the only gap junction protein present in the choroid, optic nerve, and sclera.^110^^,^^112^ *GJD2*(Cx36) is exclusively identified in the retina.

### Regulation of Expression and Phosphorylation of *GJD2*(Cx36)

The phosphorylation state of the gap junction protein Cx36 determines its coupling strength. Two phosphorylation sites have been identified: Ser110, on the intracellular loop, and Ser276 (Ser293 in mammals), on the carboxyl terminus.^134^^–^^136^ Regulation of phosphorylation is complex, differs between neuronal subtypes, and often depends on a cascade of signaling proteins. In photoreceptors (zebrafish), protein kinase A (PKA) activity can directly act on the two regulatory phosphorylation sites. PKA activation leads to phosphorylation of both residues on *GJD2*(Cx36) and subsequently causes increased gap junction coupling.^137^ In AII amacrine cells (rabbits), PKA has an indirect and opposite effect; PKA activates protein phosphatase 2A (PP2A) and ultimately dephosphorylates Ser293 on *GJD2*(Cx36). Protein phosphatase 1 can subsequently counteract this phosphorylation by inhibiting PP2A.^138^ Despite the difference in initiation, phosphorylation of *GJD2*(Cx36) is strongly correlated to intercellular coupling thereby increasing gap junction function in both photoreceptors and AII amacrine cells.^137^^–^^139^

*GJD2*(Cx36)-mediated coupling is influenced by the circadian rhythm and light exposure and is modulated by dopaminergic signaling. Again, this differs between neuronal subtypes; in AII amacrine cells, dopamine D1-like receptor (includes subtypes D1R and D5R) activation reduces coupling by increased PKA activity, via the before mentioned cascade.^138^^,^^140^^,^^141^ The AII amacrine network in mice is relatively uncoupled under scotopic illumination but is increasingly coupled by shifting to mesopic illumination and then uncoupled again under photopic conditions.^98^^,^^142^^,^^143^ This coupling modulation affects the size of receptive fields and improves the signal-to-noise ratio of the AII amacrine network by averaging the uncorrelated noise.^98^^,^^142^^,^^143^ In both ganglion and photoreceptor cells, dopamine D2-like receptor (includes subtypes D2R and D4R) activation uncouples the cells.^76^^,^^144^^,^^145^ During the day and under light exposure, increased dopamine release activates D2-like receptors, which subsequently suppresses activity of adenylyl cyclase, lowers cAMP levels, and PKA activity, ultimately uncoupling the photoreceptors.^139^ During the night, decreased D2-like receptor activation results in increased photoreceptor coupling, allowing cones to receive dim light signals from rods, which facilitates the detection of large dim objects.^145^

Apart from dopamine, adenosine is also an important modulator of coupling but works opposite from dopamine. Adenosine achieves high levels during night under scotopic conditions and achieves low levels during day under photopic conditions.^146^ Elevated adenosine levels activate adenosine A2a receptors, which highly increases photoreceptor coupling during this phase. Additionally, A1 receptors with a higher affinity for adenosine activated by low day levels, suppress adenylyl cyclase and reinforce D4 receptors to uncouple photoreceptors during the day.^135^^,^^139^^,^^145^^,^^147^

In addition to phosphorylation, *GJD2*(Cx36) transcript and protein expression are also affecting coupling strength and are under circadian control. Circadian control of *GJD2*(Cx36) protein expression is dependent on melatonin, whereas the circadian regulation of *GJD2*(Cx36) transcript expression may be controlled directly by the circadian clock.^148^ Because the SNPs associated with refractive error at the *GJD2*(Cx36) locus are intergenic and most likely influencing regulation of expression (section: *GJD2*(Cx36) - Lessons learned from studies in Humans), it could be possible that these SNPs affect the regulation of *GJD2*(Cx36) expression and transcription, which in turn could influence visual processing during different times of the day.

## *GJD2*(Cx36) in Animal Models

To uncover the biological mechanisms that lie at the basis of the GWAS findings, functional studies are warranted. In order to assess a functional role of *GJD2*(Cx36) in myopia in animal models, it is relevant to investigate the degree of conservation across species. In this section, we selected the most commonly used species for myopia research and performed a phylogenetic and conservation assessment of *GJD2*(Cx36) (see Fig. 1). In this section, we particularly focus on mice and zebrafish because of their amenability to genetic manipulation and discuss the advantages and limitations of these models.

### Conservation of *GJD2*(Cx36) Across Species

The phylogenetic tree in vertebrate lineages of reptiles and birds (see Fig. 1B) shows two subfamilies, *GJD2*(Cx35/Cx36) and *GJD1*(Cx34), as a result of a partial genome duplication. In mammals, the *GJD1*(Cx34) family is not present, whereas in teleost fish (e.g. zebrafish [*Danio rerio*]) up to four functional orthologs have been identified, caused by an additional duplication event.^149^^–^^151^

The *GJD2*(Cx35/Cx36) protein, relative to the human protein, is conserved for 98% to 100% in mammalian species and for 70% to 83% in zebrafish (see Fig. 1C). Most variation is found in the intracellular loop and in the C-terminus, whereas the two phosphorylation sites are conserved in all investigated species (see Figs. 1A, 1C). We explored the conservation of the region in which the two most replicated SNPs, rs634990 and rs524952, were identified. Relative to the human region, we found an identity score of 81.1% for the rs524952 region in mice, but no match for rs524952. We found no conservation of the two SNP regions in zebrafish. Higher conservation levels were only identified in monkeys (up to 94.8%), limiting the external validity of lower mammals and vertebrates as a model for studying the functionality of these SNPs (Supplementary Table S2). Tissue- and cell-type specific *GJD2*(Cx36) knockout models, on the other hand, can be used to study entire gene effects at the cell-cell interaction level.

### Why use Mouse and Zebrafish Models?

Both mouse and zebrafish models can help unravel the effect of functional proteins on postnatal ocular development. These species have highly conserved *Gjd2*(Cx36)/*gjd1a*(cx34.1)*/gjd1b*(cx34.7)*/gjd2a*(cx35.5)/*gjd2b*(cx35.1) proteins (mouse 98%, zebrafish 70–83%; see Fig. 1) and are well-established animal models for genetic diseases. Apart from the availability of complete gene knockouts, mice and zebrafish exhibit some practical advantages, such as rapid breeding, easy housing, and an extensive toolbox for manipulating their genome.^150^^,^^152^

Compared to mice, zebrafish are able to produce a large number of offspring multiple times a week and their functional visual system develops fast. Nevertheless, given the additional duplication of the zebrafish genome and the existence of various orthologs for some genes, it may be required to investigate multiple knockout models. Challenges applicable to both animal models include absence of a fovea, the limited visual acuity, the lack of accommodative reflex, and the small eye size. Even though hyperopic refractions have been reported for both mice and zebrafish,^153^^,^^154^ studying the relative differences between wildtype and knockout animals will provide an indication of the relation between axial length changes and refractive error. Therefore, assessment of ocular biometry (i.e. axial length and vitreous chamber depth) could be considered as the most relevant outcome when studying myopia in these models. Mice are nocturnal animals, which complicates translation of findings regarding circadian rhythm to humans. In contrast, zebrafish are diurnal species with cone-dominant vision, similar to humans.^155^^,^^156^ As a sequel of this research, we have recently explored the role of *GJD2*(Cx36) in zebrafish. Depletion of *gjd2a*(cx35.5) leads to hyperopia and electrophysiological changes in the retina and a lack of *gjd2b*(cx35.1) leads to nuclear cataract and triggered axial elongation.^154^

## Potential Mechanisms by which *GJD2*(Cx36) Contributes to Myopia

**Figure 4.**
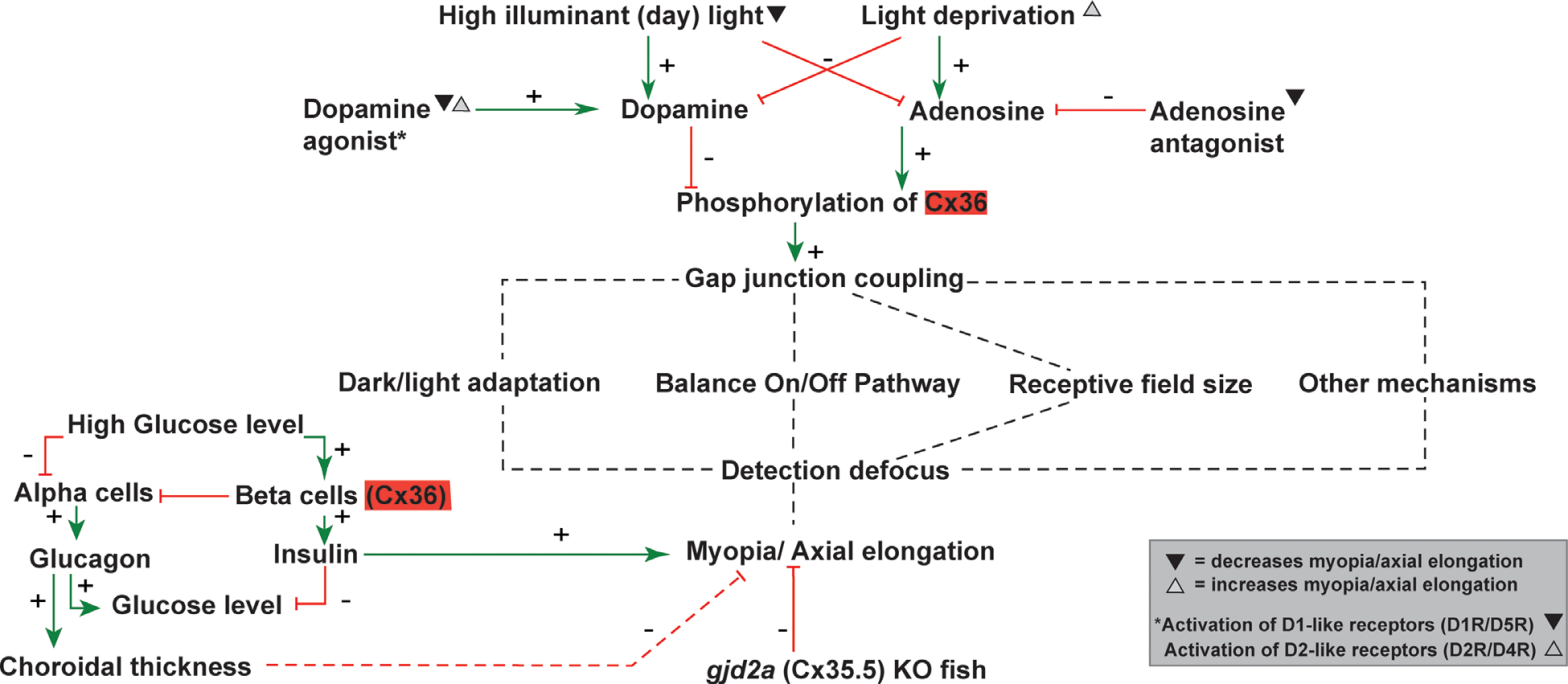
**Overview of potential mechanisms for GJD2(Cx36) (marked red) causing myopia.** Green arrows indicate a positive (i.e. stimulatory) effect; red arrows a negative (i.e. inhibiting) effect. Dotted lines indicate hypothesized mechanisms not fully advocated yet by the literature but mentioned in the current manuscript as possible mechanisms. Conditions highlighted with black triangles are frequently linked in the literature with decreased myopia/axial elongation, whereas white triangles are associated with increased myopia/axial elongation.

Various lines of reasoning, as discussed in the section *GJD2*(Cx36), suggest that the association of the *GJD2* locus in refractive error development points toward altered regulation of *GJD2*(Cx36) expression (see Table 3, Supplementary TableS1). In this section, we discuss and describe potential mechanisms by which *GJD2*(Cx36) may contribute to the pathogenesis of refractive error (see Fig. 4 for an overview of the potential mechanisms).

### *GJD2*(Cx36) Coupling and Myopia

A limited number of studies have directly linked phosphorylation of *GJD2*(Cx36) and thus, *GJD2*(cx36)-mediated intercellular coupling with myopia. A study investigating form-deprivation myopia (FDM) in chicks found that a nonspecific gap junction blocker (meclofenamic acid) diminished myopia.^157^ An FDM study in mice demonstrated increased phosphorylation of *GJD2*(Cx36)-containing gap junctions between AII amacrine cells after myopia induction. The authors suggested that the increase of phosphorylation is a compensatory effect of the defocused image. Although this fits the hypothesis, more verification is needed.^158^

Above, we mentioned the opposite coupling effects of dopamine and adenosine in response to light conditions (section: Regulation of expression and phosphorylation of *GJD2*(Cx36)).^76^^,^^132^^,^^138^^,^^140^^,^^141^^,^^144^^,^^145^ An established environmental risk factor is time spent outdoors, which offers protection against childhood myopia most likely because of increased light intensities of broad spectrum.^5^ This relation is supported by animal experiments which showed that high intensity lighting can reduce FDM in chicks and monkeys^159^^–^^161^ and LIM in mice.^162^ Proof that dopamine can be a mediator in this relationship comes from experiments in chickens showing that dopamine blockers abolish the protection by light.^163^^,^^164^

Correspondingly, pharmacological stimulation of dopamine signaling (via e.g. nonspecific dopamine receptor agonist apomorphine) protects against FDM in a wide range of species including mice,^165^^–^^167^ primates,^168^ chicks,^169^^,^^170^ guinea pigs,^171^^,^^172^ and rabbits.^173^ Conversely, dopamine receptor antagonists facilitate myopia development induced by FDM. Antagonists, per se, are not sufficient to induce myopia without external triggers.^174^^,^^175^ The FDM-protective effect of dopamine agonist (apomorphine) is nullified by simultaneous administration of D2R antagonist in chickens.^169^ Interestingly, adenosine antagonists appear to be protective against childhood and experimental myopia.^176^^–^^179^ Taken together, these findings strongly suggest that the protective effect of outdoor exposure against myopia arises from increased dopamine levels and decreased adenosine levels, which may lead to *GJD2*(Cx36) dephosphorylation and subsequent uncoupling of retinal neurons.

When elucidating the role of dopamine in myopia, it is essential to make a distinction between dopamine D1-like (subtypes D1R and D5R) and D2-like receptor (D2R and D4R) activation. Zhou et al. described opposing results on myopia development when activating and inactivating D1-like and D2-like receptors separately.^180^ They propose that emmetropization is a homeostatic process controlled by opposing effects of D1-like and D2-like receptors; pharmacological activation of D1-like receptors results in hyperopia, whereas pharmacological activation of D2-like receptors results in myopia.^180^ Interestingly, D1-like receptor activation uncouples AII amacrine cells, whereas D2-like receptor activation uncouples ganglion cells and photoreceptors (section: Regulation of expression and phosphorylation of *GJD2*(Cx36)). The disbalance between coupled AII amacrine cells versus coupled ganglion cells and photoreceptors and their relation to myopia is intriguing and may be solved by future studies.

### Receptive Field

The size of the receptive field of photoreceptors can be changed by altering the extent of the gap junction coupling.^145^^,^^181^ In line with the mechanism described earlier, *GJD2*(Cx36) mediated coupling between photoreceptors increases during the night, thus leading to larger receptive fields.^145^^,^^181^ In myopes, an increased receptive field size has also been demonstrated.^182^ Although this finding needs to be proven, this increase is likely the result of increased coupling between photoreceptors, linking coupling to myopiagenesis.^182^

### ON and OFF Signaling Pathway

*GJD2*(Cx36)-containing gap junctions connect AII amacrine cells to ON cone bipolar cells and thereby provide a signaling pathway from rods feeding into the cone pathway via rod bipolar cells (section: Expression of *GJD2*(Cx36) in the retina and the various functions in visual processing) and AII amacrine cells to ON/OFF cone bipolar cells. This *GJD2*(Cx36)-connection enables the ON pathway to cross-inhibit the OFF pathway, involving OFF bipolar and OFF ganglion cells,^183^ which could improve the efficiency of contrast encoding.^184^

Experimental settings in which elements of this pathway are disrupted provide further insights into the role of *GJD2*(Cx36) in myopia development. ERG of *GJD2*(Cx36) knockout mice showed a reduced scotopic b-wave, suggesting deficits of the rod signal pathway.^26^^,^^86^^,^^87^ In addition, patients with congenital stationary night blindness (CSNB1), who also develop high myopia, exhibit defects in the ON pathway. Imbalance of the ON and OFF pathway in causing myopia is confirmed by studies showing that ON pathway deficiency triggers myopia in mice and chickens and OFF pathway deficiency inhibits myopia in chickens, whereas a mouse study did not support this converse effect of OFF-pathway deficiency.^153^^,^^185^^–^^187^

In human subjects, overstimulated OFF pathways (1 hour of either reading black text on a white background or exposing to dynamic OFF stimuli) results in a thinner choroid and overstimulated ON pathways (1 hour of either reading white text on a black background or exposing to dynamic ON stimuli) leads to a thicker choroid.^188^^,^^189^ Chicken experiments showed similar results and demonstrated increased dopamine release during ON stimulation.^189^ Because thinner choroids are associated with myopia development and thicker choroids are associated with myopia inhibition,^190^^–^^194^ dopamine, *GJD2*(Cx36), ON and OFF pathway, and choroidal thickening seem to be tightly linked in myopia development.^195^

### Insulin and Glucagon

*GJD2*(Cx36) is expressed in the islets of Langerhans in the pancreas (as shown in Table 1) and provides electrical and metabolic coupling between beta-cells in these islets. When glucose levels are high, *GJD2*(Cx36) coordinates the synchronization of electrical activity throughout the islet, which results in pulsed secretion of insulin from beta-cells and conversely for low glucose levels.^196^ Insulin release is in anti-phase with glucagon secretion from pancreatic alpha-cells.^197^ A human exonic variant of *GJD2*(Cx36) exhibits postnatal reduction of *GJD2*(Cx36) islet levels and beta cell survival, resulting in glucose intolerance in transgenic mice.^198^

Several studies have identified an association between metabolic control of glucose (insulin/glucagon pathways) and myopia in humans.^199^^–^^202^ Interestingly, insulin and glucagon show opposing effects on eye growth in chickens, with glucagon mostly increasing choroidal thickness (associated with myopia inhibition) and insulin mostly increasing ocular elongation, proposed to be controlled by glucagon-positive amacrine cells.^203^^–^^206^

Together, these findings indicate a link between *GJD2*(Cx36) and metabolic control of glucose levels via insulin and glucagon, which have a causal effect on eye growth in chickens. Future studies exploring the effect of common variants annotated to *GJD2*(Cx36) on insulin/glucagon levels and the subsequent potential impact on myopia development may help to elucidate this potential mechanism. Furthermore, given that the glucagon-positive amacrine cells are up to now only found in the avian retina, there might be an equivalent cellular mechanism present in humans that is closely related to the glucagon sensitive system found in chickens.

## Future Directions and Conclusions

GWASs have provided insights into the genetic architecture of refractive error. However, to further elucidate the biology underlying GWAS results, follow-up studies are required. These studies should include exploring the effect of the associated variants at the 15q14 locus on gene expression levels in the retina and other regulatory mechanisms like methylation. The variants may not be directly causally involved but could also change the function of an intergenic regulatory region. It is worth noting that current insights (see Fig. 4) point to an upregulation (either at protein level or phosphorylation state) of *GJD2*(Cx36) or an effect on the circadian regulation of *GJD2*(Cx36) expression as potential mechanisms contributing to myopia.

Another next step is to functionally explore the role of *GJD2*(Cx36) in myopia development. As outlined in the section Why use mouse and zebrafish models?, mice and zebrafish are suitable models due to the availability of an extensive toolbox that allows genetic manipulation and their suitability to study the visual system. In addition to the study of *GJD2*(Cx36) knockout on the phenotype, single cell-RNA sequencing can help dissect the differential transcriptomic profile of retinal cells and thereby allow a better understanding of the visual pathway and of myopia. Limitations of animal models include the low conservation of the regulatory sequence (section: Conservation of *GJD2*(Cx36) across species). However, cell culture models or the upcoming organoids of human tissue may be useful tools to test the regulatory function of the identified variants.

In conclusion, *GJD2*(Cx36) is a major candidate gene for non-syndromic myopia. As summarized and discussed in this review, it is involved in various processes that could potentially influence the risk of myopia. Future studies focusing on disentangling the myriad functions of *GJD2*(Cx36) in the described systems might be challenging, but at the same time they are critical to shed light on the mechanisms leading to myopia. Unraveling these mechanisms may potentially generate new targets for intervention and stop the global myopia boom.

## Literature Search

We searched the PubMed database for articles without any date restrictions using the following search terms separately or in combination: “gap junction delta-2,” “connexin36,” “gap junctions,” “connexins,” “myopia,” “refractive error,” “emmetropization,” “retina,” and “ocular tissue.” In addition, a manual search was based on references from retrieved articles. Articles were excluded if they were not peer-reviewed.

## Supplementary Material

Supplement 1
